# Research progress on molecular biomarkers of acute myeloid leukemia

**DOI:** 10.3389/fonc.2023.1078556

**Published:** 2023-02-07

**Authors:** Pei-Yuan Yin, Rui-Wen Wang, Rui Jing, Xing Li, Jing-Hua Ma, Kai-Min Li, Hua Wang

**Affiliations:** ^1^ Hematology Department, Yantai Affiliated Hospital, Binzhou Medical University, Yantai, Shandong, China; ^2^ Department of Blood Supply, Yantai Center Blood Station, Yantai, Shandong, China; ^3^ Department of Anesthesiology, Yantai Yuhuangding Hospital, Qingdao University, Yantai, Shandong, China; ^4^ Department of Science and Education, Yantai Hospital of Traditional Chinese Medicine, Yantai, Shandong, China; ^5^ Hematology Department, Yantai Yuhuangding Hospital, Qingdao University, Yantai, Shandong, China

**Keywords:** acute myeloid leukemia, biomarker, gene mutation, chromosome, epigenetics

## Abstract

Acute myeloid leukemia (AML) is the most common type of adult acute leukemia. The pathophysiology of the disease has been studied intensively at the cellular and molecular levels. At present, cytogenetic markers are an important basis for the early diagnosis, prognostic stratification and treatment of AML. However, with the emergence of new technologies, the detection of other molecular markers, such as gene mutations and epigenetic changes, began to play important roles in evaluating the occurrence and development of diseases. Recent evidence shows that identifying new AML biomarkers contributes to a better understanding of the molecular mechanism of the disease and is essential for AML screening, diagnosis, prognosis monitoring, and individualized treatment response. In this review, we summarized the promising AML biomarkers from four aspects, which contributing to a better understanding of the disease. Of course, it must be soberly aware that we have not listed all biomarkers of AML. Anyway, the biomarkers we mentioned are representative. For example, mutations in TP53, FLT3, and ASXL1 suggest poor prognosis, low remission rate, short survival period, and often require allogeneic hematopoietic stem cell transplantation. The CEBPA double mutation, NPM1 and CBF mutation suggest that the prognosis is good, the remission rate is high, the survival period is long, and the effect of chemotherapy or autotherapy is good. As for other mutations mentioned in the article, they usually predict a moderate prognosis. All in all, we hope it could provide a reference for the precise diagnosis and treatment of AML.

## 1 Introduction

Acute myeloid leukemia (AML) is a type of malignant tumor affecting hemopoietic stem cells/progenitor cells and is characterized by abnormal proliferation of primitive cells in bone marrow and peripheral blood. The clinical manifestations of AML include anemia, hemorrhage, infection and organ infiltration. The incidence of AML has been increasing with the passage of time, and men are more likely to suffer from the disease than women (1). At present, cytogenetic and molecular abnormalities are still the most important prognostic factors of AML and are closely related to clinical features (age, white blood cell count and morphology), treatment response, recurrence rate and overall survival (OS) (2–4). This has laid a solid foundation for the development of disease prognostic stratification by the World Health Organization (WHO) and European Leukemia Net (ELN). At present, the five-year survival rate of all adult AML patients is less than 50%, which is even lower in elderly patients. Statistics have shown that the median OS of patients > 65 years is less than one year (5). This paper summarizes the molecular biomarkers related to AML and elaborates their clinical value in early diagnosis and prognosis. Among them, prognostic markers can estimate the severity of the disease and predict the long-term outcome of patients, whereas predictive markers allow the surveillance of treatment response and recurrence.

## 2 Marker source 1: Specific gene mutation-related molecules

### 2.1 Nucleophosmin 1

NPM1 mutation is the most common gene mutation related to AML. NPM1 is located in the long arm of chromosome 5 and encodes a multifunctional chaperone protein shuttling between the nucleus and cytoplasm. NPM1 is mainly involved in the regulation of ribosomal protein assembly and trafficking, the stabilization of the tumor suppressors p19ARF and p53 pathway, DNA repair process, genome stability, and ultimately regulates DNA transcription by altering chromatin structure. As a tumor suppressor, any mutation that disrupts its normal function will lead to the transformation of normal cells into malignancy. In addition, wild-type NPM1 protein localizes to the nucleolus in normal cells. However, the mutant protein usually mislocalizes to the cytoplasm, which is closely related to the pathogenesis of leukemia, but its specific mechanism is still unclear (6). NPM1 mutations are only found in myeloid cells and have been detected in a few cases of chronic myelomonocytic leukemia, all of which progressed to AML within one year. NPM1 mutations are most frequently detected in the M4 and M5 subtypes of AML but are rarely found in acute promyelocytic leukemia (APL). AML accounts for approximately 30% of all AML cases and 40-60% of AML cases with normal karyotypes. Clinical data showed that patients with mutant NPM1 had a higher number of myeloid progenitor cells, higher white blood cell and platelet counts, lower CD34 level, a complete remission rate (CRR) of 58-60%, and a median OS of 16.2 months (6).

ELN guidelines suggest that NPM1-mutant patients with an allelic ratio (AR) < 0.5 for FMS, such as tyrosine kinase 3-internal tandem replication (FLT3-ITD), have a good prognosis, and allogeneic hematopoietic stem cell transplantation (allo-HSCT) is not recommended after the first complete remission. According to the 2021 National Comprehensive Cancer Network (NCCN) guidelines, NPM1-mutant patients without FLT3-ITD or with FLT3-ITDlow are classified into the good prognosis group, whereas patients with NPM1 mutation and FLT3-ITDhigh are classified into the intermediate prognosis group. Therefore, it is assumed that leukemogenesis is not induced by NPM1 mutation alone but is also related to other cellular and molecular genetic changes, such as FLT3-ITD.

It has been found that NPM1 mutation accompanied by mutations in isocitrate dehydrogenase 1/2 (IDH1/2) and DNA methyltransferase 3 alpha (DNMT3A) led to poor prognosis, and it is generally believed that such mutations are secondary to the NPM1 mutation. Similar to promyelocytic leukemia-retinoic acid receptor α (PML-RARA) rearrangement, NPM1 can also serve as a marker for the surveillance of minimal residual disease (MRD) in AML patients. That is, the increase in mutant NPM1 in peripheral blood is predictive of the relapse of AML in patients with complete remission morphologically. Therefore, timely and reasonable intervention can be carried out according to the NPM1 level in MRD. For AML patients with NPM1 mutation, it is currently known that combined application of all-trans retinoic acid (ATRA) and arsenic trioxide (ATO) can degrade the mutant NPM1 protein and further induce the apoptosis of AML cells (7). Our understanding of the mechanism of targeted therapies based on NPM1 is still incomplete, and large-scale basic studies are still needed to explore the function and mechanism of NPM1 to provide a new theoretical basis for the treatment of AML.

### 2.2 FLT3

FLT3 is located on the long arm of chromosome 13 and is a member of the type III receptor tyrosine kinase (RTK) family. It participates in the proliferation, differentiation and apoptosis of hematopoietic cells through the extracellular domain containing ligand binding sites. FLT3 mutation is the most common RTK mutation in AML, accounting for approximately 30% of cases, of which ITD accounts for approximately 25%, and tyrosine kinase domain (TKD) point mutation accounts for only 7%. FLT3-ITD is usually common in AML with a normal karyotype, and the prognosis is poor. At the molecular level, mutant FLT3 activates downstream factors related to the STAT5, RAS/MAPK and PI3K signaling pathways.

In terms of clinical characteristics, relevant statistical data showed that the expression level of FLT3 was correlated with different National Comprehensive Cancer Network(NCCN) stratification of AML, with the lowest level in the M3 subtype and the highest level in M5. Several studies have shown that patients with FLT3 mutations are mainly characterized by high white blood cell counts in the peripheral blood, high myeloid progenitor cell counts, and poor prognosis (8–10). Among them, FLT3-ITD patients are prone to combined PML-RARA rearrangement, which rarely occurs in AML patients with complex karyotypes or core binding factor (CBF)-AML. Compared with FLT-TKD patients, FLT-ITD patients have significantly lower OS and event-free survival (EFS) and worse prognosis. A study showed that the prognosis of FLT3-mutant patients was closely correlated with the expression ratio of the mutant FLT3 allele against the wild-type FLT3 allele. The greater the ratio, the shorter the duration of remission (DOR), EFS and OS. Therefore, DNA fragment analysis technology was utilized to quantify the relative level of mutant alleles and to define a cutoff value to distinguish different prognostic subgroups (11).

For AML with FLT3 mutation, small molecule tyrosine kinase inhibitors such as sorafenib, midostaurin and sunitinib have been available for the inhibition of the FLT3 signaling pathway and for the targeted killing of leukemia cells. In recent years, a large number of clinical trials have shown great improvements in the therapeutic effect of multitarget small molecule tyrosine kinase inhibitors on AML patients compared with traditional chemotherapy. However, due to drug resistance, the DORs of such drugs alone are still not satisfying, and combination use with other chemotherapy drugs has become the preferred strategy for the treatment of this type of AML.

### 2.3 Tumor protein p53

TP53 is located in the short arm of chromosome 17 and encodes a key transcription factor, which is mainly involved in DNA mismatch repair, base excision repair and nucleotide excision repair at the time of cell cycle arrest. When this tumor suppressor gene is mutated, such as with a deletion mutation, cell proliferation becomes uncontrolled, and tumorigenesis may occur. TP53 mutations exist in a variety of cancers, such as Li-Fraumeni syndrome, ovarian cancer, esophageal cancer, colorectal cancer and lung cancer, and their mutation frequency is associated with cancer type. The IARC TP53 database shows that TP53 mutations are not as common in hematological malignancies as in other types of cancer (approximately 5.89%, compared with 12.77% in colorectal cancer and 10.2% in breast cancer). In AML, most TP53 mutations involve single nucleotide changes, with missense mutations being the most common, followed by frameshift mutations and nonsense mutations. TP53 mutations account for approximately 10% of newly diagnosed AML, 20-37% of therapy-related AML (T-AML) or secondary AML, and up to 70% of complex karyotype AML. They are important markers of poor prognosis in complex karyotype AML and T-AML. TP53 mutations are more common in elderly patients, patients with 17p mutation or chromosome 5 and 7 aberrations, with significantly lower CRR, higher recurrence rate, and shorter EFS and OS. Compared with patients with wild-type TP53, the CRR of TP53-mutant patients was only 28.6%. After chemotherapy with cytarabine or demethylating drugs, the patients’ median OS was 6-8 months. Even after HSCT, such patients will still have a high recurrence rate and serious adverse reactions, with a median OS of 8 months, a one-year survival rate of 35%, and a recurrence rate after one year of 53% (12). Recently, a study showed that TP53-mutant patients were possibly more sensitive to the 10-day regimen of decitabine chemotherapy, and the CRR of the mutant TP53 group was significantly better than that of the wild-type TP53 group (13). The ELN guidelines recommend early screening of TP53 mutation to assist in prognostic stratification and making initial treatment plans. At present, the treatment options for patients with TP53 mutations are limited, and the poor prognosis of standard chemotherapy regimens may be related to resistance to chemotherapy drugs. In 2018, the American Society of Hematology proposed that the therapeutic response of the new TP53 regulator APR-246 was significantly better than those of traditional regimens, with a CRR of approximately 82% and a median OS of more than 7 months. The European Medicines Agency approved APR-246 as an orphan drug for the treatment of myelodysplastic syndromes (MDS), AML and ovarian cancer. The Food and Drug Administration (FDA) also approved APR-246 combined with azacitidine for the treatment of Li-Fraumeni syndrome complicated with MDS in January 2020. A recent study showed that APR-246 and azacitidine play a synergistic role in TP53-mutated MDS/AML, which can restore the transcriptional activation function of mutant TP53 and induce apoptosis of human tumor cells (14), and preclinical trials are currently underway.

### 2.4 CCAAT enhancer binding protein alpha

The CEBPA gene is located on the long arm of chromosome 19. CEBPA protein is a transcription factor with leucine zipper, the structure of which includes the transcriptional active region at the N-terminus, the DNA-binding region, and the leucine rich dimerization functional region at the C-terminus. There are two types of CEBPA mutations: out-of-frame insertions or deletions at the N-terminus and in-frame insertions or deletions at the C-terminus. CEBPA double mutation (biCEBPA) refers to the mutation of CEPBP on both alleles of chromosome 19. CEBPA plays a key role in the differentiation of hematopoietic myeloid cells. Approximately 10% of AML patients and 7-15% of AML patients with normal karyotypes carry CEBPA mutations. Most of them are the M2 type, and their prognosis is generally good. According to the 2016 WHO classification of hematopoietic and lymphoid neoplasms, “AML with biCEBPA” was defined as a clinical subtype of AML with unique molecular biological characteristics. The ELN guidelines also recommend early screening of CEBPA mutations. Multicenter clinical data showed that compared with all AML patients, biCEBPA was significantly correlated with improved EFS and OS when receiving cytarabine-based chemotherapy, with hazard ratios (HR) of 0.41 and 0.37, respectively, but was not correlated with consolidation strategies such as HSCT, indicating that biCEBPA was positively correlated with improved prognosis. Interestingly, through sequencing analysis of normal karyotype AML patients with CEBPA mutations in recent years, researchers have found that Tet methylcytosine dioxygenase 2 (TET2) and GATA2 mutations are more likely to occur in biCEBPA patients (approximately 30%), while CEBPA single mutation (moCEBPA) patients are more likely to have combined NPM1, FLT-ITD/TKD and IDH2 mutations, and the prognosis is relatively poor when combined with TET2 mutations (15, 16). Although CEBPA has not yet been identified as a marker of MRD, the specific immunophenotype associated with biCEBPA might become a favorable tool for disease screening and treatment monitoring. Here, what needs to be specially mentioned was GATA2 mutation. GATA2 may be a novel susceptibility gene for familial AML (17). GATA2 is considered to be a hematopoietic “stem” gene, which is highly expressed in hematopoietic stem cells and is required for megakaryocyte and mast cell generation. GATA2 is down-regulated during myeloid differentiation, forcing over-expression to inhibit this differentiation. The discovery of GATA2 mutant in AML susceptibility family provides a new method to explore the mechanism of GATA2 inducing leukemia, and may clarify its role in maintaining “stem”. The prognosis of AML was poor when the patient with GATA2 mutations. Boldly, it may be appropriate to take active strategies to treat the affected individuals in the family with GATA2 mutations before symptoms.

## 3 Marker source 2: Chromosome position abnormality-related molecules

### 3.1 PML-RARA

PML-RARA is encoded by a fusion gene generated by the t (14; 16) (q24; q21) translocation. It is a major molecular feature of APL and is present in approximately 98% of APL patients (18). PML-RARA plays a role in causing APL by two primary effects, which is deregulates transcriptional control (19). PML is mainly involved in the regulation of signaling pathways and induction of the transcription of the cell cycle inhibitor p27Kip and the proapoptotic factor Bim. The RARA gene is located on 17q21 and encodes a nuclear receptor that activates transcription in the presence of its ligand retinoic acid, inducing many target genes involved in differentiation. RARA and retinoic acid X receptor α (RXRA) form the heterodimer RAR-RXR, which constitutes a transcriptional activator complex required for promyelocyte differentiation (20). In the absence of retinoic acid, RAR-RXR acts as a transcriptional repressor by recruiting the accessory repressors DNMT1, DNMT3A, histone deacetylase and histone methyltransferase and participates in chromatin remodeling. In the presence of retinoic acid, RAR-RXR undergoes a conformational change, which leads to the dissociation of RAR-RXR and the activation of genes required for primitive cell differentiation. Fusion of the PML and RARA proteins can disrupt this coactivator recruitment to prevent transcription of retinoic acid response elements (21). APL accounts for approximately 10-15% of all AML cases. Low-risk APL patients can achieve a CRR of 100% and a 2-year EFS of 97% after receiving ATRA combined with ATO. In APL patients, the presence of PML-RARA rearrangement suggests a good response to ATRA and other retinoids, and the combination of ATRA and chemotherapy significantly improved the survival rate of patients (22–24). Therefore, PML-RARA screening for patients with suspected APL is of great value for the optimization of patient management and for etiological research. PML-RARA mutations can be detected by fluorescence *in situ* hybridization (FISH) or real-time quantitative polymerase chain reaction (RT−qPCR), which can not only assist in diagnosis and treatment and evaluate therapy efficacy but also monitor the changes in MRD, which is of great significance in monitoring recurrence, estimating prognosis and determining the time of drug withdrawal. However, to date, more than ten fusion genes with different counterparts of RARA have been found in roughly 1% to 2% of APL patient, contains ZBTB16-RARA(PLZF-RARA), which is the most frequent APL molecular variant (25, 26), and other translocations led to the rearrangement of RARA gene with NPM1, NUMA1, STAT5B, PRKAR1A, FIP1L1, BCOR, NABP1, TBL1XR1, GTF2I, IRF2BP2, and FNDC3B, etc, most of them were non-sensitive to ATRA and ATO (27). So if such fusion genes were detected in APL patients, which indicated that they will not have a good treatment effective to ATRA and ATO.

### 3.2 CBF

CBF mutations in AML are cytogenetically characterized by t (8; 21) or inv (16)/t (16; 16), producing RUNX1-RUNX1T1 (AML1-ETO) or CFB β subunit-myosin heavy chain 11 (CBFβ-MYH11) fusion proteins, respectively (28). CBF mutations are one of the most common cytogenetic mutations in AML patients, accounting for approximately 30% of pediatric AML and 15% of adult AML cases. Relevant studies have shown that AML1-ETO and CBFβ-Myh11 alterations on their own are not sufficient to induce leukemia. For the pathogenesis of CBF-AML, the “double hit” model is widely recognized at present, in which the molecular genetic alterations of AML1-ETO, CBFβ- MYH11, CCND1, and CCND2 play important roles (29, 30).

The t (8; 21) (q22; q22) translocation is common in leukemia and generates an AML1-ETO fusion protein. The AML1 (RUNX1) protein family is also known as a CBFα. It consists of a group of heterodimeric transcription factors, which consist of an α subunit (CBFα) encoded by 3 different genes RUNX1/RUNX2/RUNX3 and a β subunit (CBFβ) encoded by CBFβ. At the molecular level, RUNX1 is a key transcription factor during hematopoietic cell differentiation and myeloid development, whereas ETO (RUNX1T1) assists transcriptional repression mainly by recruiting corepressors. AML1-ETO, as a transcriptional repressor, directly blocks the transcription of AML1-dependent tumor suppressors, disrupts normal hematopoietic cell differentiation, and promotes leukemia progression. In addition, it also inhibits the activity of hematopoietic transcription factors such as PU1, GATA1, and CEBPA and thus disrupts the normal hematopoietic process. T (8; 21)-positive AML accounts for 5-10% of all AML cases, including 7-12% of adult AML cases. It is common in the AML-M2 subtype but rare in the M1 and M4 subtypes. The clinical characteristics are generally better prognosis, higher remission rate and longer median OS. The most common gene mutation in AML, c-Kit, which accounts for 20-25% of newly diagnosed cases, is one of the important synergistic factors in the pathogenesis of t(8;21)-positive AML. According to the NCCN guidelines, CBF-AML with c-Kit mutation is classified into the intermediate prognosis group.

Chromosome 16 inversion inv (16) (p13; q22) produces the fusion gene CBFβ-MYH11, which encodes the CBFβ-MYH11 fusion protein. As a transcriptional repressor, CBFβ-MYH11 cooperates with AML1 in the transcriptional inhibition of PTEN, Bcl-2, CEBPA, ARF and PSGL-1. Normally, CBFα and CBFβ form a CBFα/CBFβ complex on DNA at the RUNX binding site and regulate gene expression. When gene rearrangement occurs, the C-terminus of MYH11 fuses with the CBFβ residue. As the CBFβ-MYH11 fusion protein is the isomeric to RUNX1 (CBFα), it interrupts the normal function of the CBFα/CBFβ complex through competitive inhibition. CBFα is a key regulator in hematopoiesis. Abnormal fusion products will damage its normal function and inhibit hematopoietic cell differentiation. At present, RT−qPCR and FISH are sensitive and effective measures to detect these rearrangement mutations (31, 32). AML patients with inv (16) generally have good prognosis and better response to conventional chemotherapy (combination therapy with anthracyclines and cytarabine, the so-called “3+7” regimen). In recent years, with the development in cytogenetics and molecular genetics, researchers have found various gene mutations and chromosomal abnormalities related to CBF-AML; the former includes mutations in FLT3, TET2, JAK2V617F, ASXL1, RAS and CBL, and the latter includes sex chromosome deletion, chromosome, 9q-, +8, +4, and chromosome 7 long arm abnormalities. Among them ASXL1 mutation is worth mentioning (33). ASXL1 mutation in patients with hematologic malignancies was first reported in 2009. After that, researchers gradually found that approximately 6% to 30% of patients with AML had ASXL1 mutations, in particular older patients and patients with secondary were the two most common victims. Additionally, ASXL1 mutations adversely affected the survival of patients with AML. For example, it could accelerate the conversion of MDS to AML. So, only by continuous in-depth study of the pathogenesis of CBF-AML can accurate and individualized treatment of these patients be achieved.

### 3.3 Histone lysine methyltransferase 2A (KMT2A)

KMT2A is located in region 2 band 3 of the long arm of human chromosome 11 (11q23). It is a member of the trithorax group (TrxG) family. Rearrangement of KMT2A includes translocation, deletion, insertion, inversion and partial tandem duplication, of which translocation is the most common, which was first reported by Ziemin-van der Poel et al. in 1991 (34). In 2002, Armstrong et al. (35) reported that KMT2A encodes a transcription coenzyme that regulates gene expression during early embryonic development and hematopoietic cell differentiation. It contains an SET region with methyltransferase activity, which methylates the lysine residue at the fourth position of histone H3, thereby activating the transcription of homeotic genes (HOX). KMT2A is widely expressed in hematopoietic cells, including stem cells and progenitor cells. It has leukemogenic effects only after fusing with a variety of partner genes, such as AF4, AF9, ENL, AF10 and ELL (36). When MEN1 and LEDGF bind to the N-terminus of KMT2A, they further activate HOXA9 and HOXA10, which are usually upregulated in leukemia with KMT2A mutation. KMT2A rearrangement accounts for 43-58% of cases in infant AML, 39% in children < 2 years, 8-9% in children > 2 years, and approximately 5% in adult AML (37). In general, the incidence of KMT2A rearrangement decreases with age and is approximately four times higher in children than in adults. Approximately 5-10% of topoisomerase II inhibitor therapy-associated AML cases are accompanied by KMT2A rearrangements. These patients usually have a poor prognosis, with a low remission rate and a high chance of recurrence (38). It has been shown that allo-HSCT could reduce the relapse rate and mortality risk of AML patients with KMT2A rearrangement after remission (39). However, allo-HSCT has disadvantages, such as a lack of donors and harsh transplantation conditions. Recently, scientists have focused on the exploration of drugs targeting transcription mediated by fusion proteins. Grembecka et al. screened two small molecule inhibitors, MI-2 and MI-3, through high-throughput sequencing, which were found to interfere with the binding of MEN1 to KMT2A to release the KMT2A-AF9 fusion protein from binding to the target gene promoter (40). Other drugs targeting the fusion protein have also been developed, which is expected to provide a new strategy for the treatment of AML with KMT2A mutation.

## 4 Marker source 3: Aberrant DNA methylation and related regulatory molecules

DNA methylation is a type of epigenetic modification that regulates gene expression and plays a key role in hematopoiesis and other processes in human development. DNA methylation is catalyzed by three DNA methyltransferases, DNMT3A, DNMT3B, and DNMT1. DNMT3A and DNMT3B are responsible for initiating DNA methylation on unmodified DNA templates, whereas DNMT1 methylates newly synthesized DNA strands using the partially methylated DNA strand as templates during replication. Mutations in NPM1, CEBPA and RUNX1 can be defined by different DNA methylation levels. These genetic phenotypes are associated with DNA methylation levels. For example, DNMT3A is a required oncogenic transcription factor for PML-RARA in APL. In another study on the specific epigenetic characteristics of DNA methylation, AML patients were divided into 16 subgroups based on DNA methylation levels. These subgroups were associated with cytogenetic or molecular genetic changes, such as inv (16), t (8; 21) and PML-RARA (41). Genome-wide DNA methylation levels can also predict the clinical prognosis of AML patients. The overall DNA methylation level of AML patients at relapse was found to be lower than that at the time of first diagnosis, and a high level of demethylation was associated with improvements in prognostic indicators such as CRR and OS (42). The above studies showed that aberrant DNA methylation is an important event in the pathogenesis of AML and can serve as a powerful epigenetic marker in the early diagnosis, prognosis surveillance and treatment decision-making of AML. Of course, the regulatory molecules involved in DNA methylation aberration in AML also deserve attention.

### 4.1 TET2

TET2 is located on the long arm of chromosome 4 and encodes methylcytosine dioxygenase, which is associated with DNA demethylation. First, 5-methylcytosine is converted to 5-hydroxymethylcytosine, and then 5-formylcytosine is converted to 5-carboxycytosine. Finally, demethylation is achieved through base excision and repair mediated by DNA glycosylase. TET2 and IDH1/2 mutations are mutually exclusive and play key roles in myeloid cell differentiation, leading to abnormal hematopoietic differentiation and inducing abnormal proliferation of hematopoietic stem cells/progenitor cells. A recent study showed that TET2 mutations occur in leukemia hematopoietic stem cell precursors and are early events in leukemogenesis (43). Epigenetic studies have identified the correlation between TET2 mutations and hypermethylation in AML. The specific mechanism is that TET2 mutations lead to decreased levels of 5-hydroxymethylcytosine and induce DNA hypermethylation mainly in the enhancer regions, which are well-known key regulatory sites of tumor suppressor genes, thereby inducing DNA methylation in nearby regions and affecting gene expression. Approximately 10-20% of AML patients carry TET2 mutations (including deletion, nonsense and missense mutations), which often coexist with NPM1, FLT3, and DNMT3A mutations. At present, there is no definitive conclusion on the prognostic value of TET2 in AML patients. Some studies believe that TET2 mutations are associated with poor prognosis of AML, but others suggest that TET2 mutations have no significant impact on the prognosis of AML patients. This controversy remains to be further explored.

### 4.2 DNMT3A

DNMT3A is located on the short arm of chromosome 2 and plays an important role in DNA *de novo* methylation, catalyzing the addition of new methyl groups to cytosine residues on CpG dinucleotide sequences, thereby regulating gene transcription. DNMT3A mutations have serious impacts on DNA methylation, which leads to global changes in gene expression, accompanied by the blockage of normal differentiation of hematopoietic cells. These mutations mainly interrupt the catalytic domain of DNMT3A and lead to hypomethylation of normally overexpressed hematopoietic stem cell-specific genes (such as RUNX1, ERG, MYC, and SMAD3) in AML, thereby interfering with the normal differentiation of hematopoietic stem cells. Relevant studies have clarified that DNMT3A is required for hematopoietic stem cell self-renewal and bone marrow differentiation and is defined as an early event of AML. DNMT3A mutations induced malignant transformation of myeloid cells *in vivo*, which led to AML (44). The most common mutation of DNMT3A in AML is a missense mutation at R882 that affects the coding of an arginine and leads to the loss of methylation activity of DNMT3A. In addition, there are frameshift mutations and nonsense mutations. DNMT3A mutations are the earliest and recurrent variations in myeloid malignancies, accounting for approximately 20-22% of newly diagnosed AML in adults. DNMT3A mutations are mainly found in the M4 and M5 subtypes, with incidences of 20.5% and 13.6%, respectively. Nearly all AML patients with normal karyotypes have a single site mutation in at least one DNMT3A allele, and 30-37% of patients have loss-of-function mutations in DNMT3A. It has been clear that DNMT3A mutations have important prognostic significance for AML with a normal karyotype. Patients with these mutations generally have a worse prognosis and significantly lower OS than AML patients with wild-type DNMT3A. The hypomethylating drug (HMA) decitabine can improve the response of patients with DNMT3A mutations and achieve a higher clinical remission rate and better OS (75% vs 34% and 15.2 vs 11 months, respectively) than wild-type DNMT3A patients. High levels of mir29b targeting DNMT3A are a good marker to evaluate the therapeutic response to decitabine. Patients with myeloid malignancies harboring DNMT3A and IDH1/2 mutations showed good responses to decitabine, azacitidine, specific DNMT inhibitors, and HMAs.

### 4.3 IDH1/2

IDH1 and IDH2 are located in the long arm of chromosome 2 and chromosome 15, respectively, encoding the tumor suppressor proteins IDH1/2, which localize in the cytoplasm and peroxisomes. They are involved in intermediate metabolism and energy production in organisms. IDH1/2 promote leukemogenesis by causing DNA and histone hypermethylation, which destroys the normal differentiation of bone marrow. There are three isoforms of isocitrate dehydrogenase, namely, IDH1, IDH2 and IDH3. IDH1 is located in the cytoplasm, and IDH2 and IDH3 are located in mitochondria. Approximately 15-20% of AML cases and 25-30% of AML with normal karyotypes have IDH1/2 mutations. There was no significant difference in mutation frequency between pediatric and adult patients. IDH1/2 mutations are usually accompanied by NPM1 mutations but not FLT3-ITD mutations. Epigenetic alterations caused by IDH1/2 mutations exacerbate the proliferation of hematopoietic progenitor cells. IDH1/2 mutations inhibit histone demethylation, which is closely correlated with DNA hypermethylation, differentiation arrest and clonal expansion of hematopoietic stem cells. AML patients with IDH1/2 mutations have poor prognosis, generally with low white blood cell counts and high platelet counts, especially when accompanied by other mutations, such as NMP1 and FLT3-ITD. However, IDH1/2 mutations rarely coexist with TET2 or WT1 mutations, probably because they all affect DNA methylation (45). However, the results of relevant clinical studies on the prognostic role of IDH mutations in AML are not consistent. In NPM1-mutated AML, the combination of IDH1/2 mutations is negatively correlated with patient prognostic indicators such as relapse-free survival (RFS) and OS (46, 47). In contrast, Patel et al. reported that IDH1/2 mutations are beneficial to the survival of NPM1-mutated AML patients (48). In another study, IDH1/2 mutations were detected in 31 AML patients by second-generation sequencing, and the correlation between IDH1/2 mutations and MRD was explored. The results showed that IDH1/2 mutations were reliable MRD markers, which could predict the recurrence of most patients (49). In recent years, a large number of clinical studies have been carried out on small molecule IDH1/2 inhibitors. The newly reported small molecule inhibitor of IDH1 ivosidenib (AG-120) can inhibit the production of 2-hydroxyglutarate and promote normal cell differentiation. In 2018, the FDA approved ivosidenib for the treatment of relapsed or refractory AML with IDH1 mutations (50). Similar to ivosidenib, enasidenib (AG-221), a small molecule IDH2 inhibitor, was developed to bind IDH2 dimers and block the production of 2-hydroxyglutarate in patients with IDH2 mutations. It was also approved by the FDA in August 2017 for treating relapsed or refractory AML with IDH1/2 mutations (51). Current clinical trials mainly focus on exploring the efficacy of the combined therapy of cytarabine with the above two drugs in AML patients.

## 5 Marker source 4: Non-coding RNAs represented by miRNAs

MicroRNAs (miRNAs) are small RNA molecules composed of 17-25 nucleotides that are mainly involved in the post-transcriptional regulation of mRNA and play important roles in cell proliferation, differentiation and apoptosis. MiRNAs of different subtypes are abnormally expressed in a variety of malignant tumors with tissue specificity. In blood, miRNAs are very stable and resistant to degradation by RNase. Their expression level in blood is related to the type, stage, grade and prognosis of tumors. Ma et al. (52) showed that cytogenetically normal AML (CN-AML) patients with high miR-362-5p expression had lower OS than the control group, which implied the oncogenic function of miR-362-5p in AML. This study also suggested that miR-362-5p could be an independent poor prognostic factor for CN-AML. Zhang et al. (53) found that mir-216b overexpression was associated with poor chemotherapy efficacy and poor prognosis in AML patients and that the expression of mir-216b significantly decreased from post-chemotherapy to complete remission and significantly increased after relapse. Mir-3151 is located within the first intron of the BAALC gene (54). In a study that recruited only CN-AML patients older than 60 years, mir-3151 was identified as an independent prognostic factor (55). The study of Díaz-Beyá et al. (56) showed that AML patients with higher mir-3151 expression had shorter OS and a higher cumulative recurrence rate. Another study by this research group reported that the upregulation of mir-196b and mir-644 was associated with lower OS, and the downregulation of mir-135a and mir-409-3p was associated with higher recurrence risk (57). Lin et al. (58) showed that compared with AML patients with downregulated miR-335 expression, AML patients with upregulated miR-335 expression had shorter RFS and OS. Zhao et al. (59) showed that the expression of miR-96 in newly diagnosed AML patients was significantly downregulated and was significantly increased after treatment compared with the normal control group and that CRR was positively correlated with the miR-96 expression level. Aleksandra et al. (60) reported that the expression of miR-204 was significantly downregulated in AML patients regardless of sex and was significantly increased following successful chemotherapy (daunorubicin + cytarabine). After induction chemotherapy, AML patients with upregulated miR-204 had a higher CRR. Therefore, miR-204 is expected to become a biomarker for the prognostic evaluation of AML (61). Hu et al. (62) suggested that high miR-98 expression in AML patients who had received only chemotherapy was indicative of good prognosis. In conclusion, the above results showed that miRNAs are expected to become biomarkers for the initial screening, diagnosis and prognosis evaluation of AML, but many clinical issues still require further exploration.

## 6 Summary and prospects

As a group of hematologic malignancies with diverse biological characteristics and prognoses, AML is highly heterogenic. In this review, we summarized the promising AML biomarkers from four aspects, which contributing to a better understanding of the molecular mechanism of the disease. Of course, it must be soberly aware that we have not listed all biomarkers of AML. Anyway, the biomarkers we mentioned are representative. For example, mutations in TP53, FLT3, and ASXL1 suggest poor prognosis, low remission rate, short survival period, and often require allogeneic hematopoietic stem cell transplantation. The CEBPA double mutation, NPM1 and CBF mutation suggest that the prognosis is good, the remission rate is high, the survival period is long, and the effect of chemotherapy or autotherapy is good. As for other mutations mentioned in the article, they usually predict a moderate prognosis. As is known to clinicians, whether or not turn negative of MRD after treatment of AML is extremely important for the prognosis of patients. The group with good prognosis has a higher rate of MRD turning negative after chemotherapy, and the rate of mutation gene turning negative is also high. The group with poor prognosis has a lower rate of MRD turning negative after chemotherapy, and the rate of mutation gene turning negative is also low. So, the study of AML biomarker from cytogenetics, molecular biology and pathophysiology is conducive to the correct evaluation of its prognostic factors to achieve the precise implementation of individualized hierarchical treatment. It is believed that with the progress of experimental technology, more meaningful and novel biological indicators for AML diagnosis and treatment will be found in the future to guide personalized medicine and precise medicine to improve the survival rate and quality of life of patients. At the same time, it should be further emphasized that although the data on emerging biomarkers such as epigenetic indicators and non-coding RNAs are encouraging, we cannot conclude which marker(s) is/are the best based on current knowledge. From a clinical perspective, a set of biomarkers seems more appropriate. Therefore, it is urgently required to develop one or several reproducible and effective schemes adopting a combination of multiple biomarkers through multicenter research to achieve real clinical transformation and application.

## Author contributions

(I) Conception and design: P-YY and HW. (II) Administrative support: RJ. (III) Provision of study materials: R-WW, RJ and J-HM. (IV) Collection and assembly of data: P-YY, R-WW and K-ML. (V) Data analysis and interpretation: P-YY and HW. (VI) Manuscript writing: all authors. (VII) Final approval of manuscript: all authors. All authors contributed to the article and approved the submitted version.

## References

[1] Yi M Li A Zhou L Chu Q Song Y Wu K. The global burden and attributable risk factor analysis of acute myeloid leukemia in 195 countries and territories from 1990 to 2017: Estimates based on the global burden of disease study 2017. J Hematol Oncol (2020) 13(1):72. doi: 10.1186/s13045-020-00908-z 32513227 PMC7282046

[2] Wiatrowski K Kim TH Przespolewski A. Cellular and molecular biomarkers predictive of response to immunotherapy in acute myeloid leukemia. Front Oncol (2022) 12:826768. doi: 10.3389/fonc.2022.826768 35664748 PMC9160191

[3] Kayser S Levis MJ. Clinical implications of molecular markers in acute myeloid leukemia. Eur J Haematol (2019) 102(1):20–35. doi: 10.1111/ejh.13172 30203623 PMC7883114

[4] Prada-Arismendy J Arroyave JC Röthlisberger S. Molecular biomarkers in acute myeloid leukemia. Blood Rev (2017) 31(1):63–76. doi: 10.1016/j.blre.2016.08.005 27639498

[5] Riva L Luzi L Pelicci PG. Genomics of acute myeloid leukemia: The next generation. Front Oncol (2012) 2:40. doi: 10.3389/fonc.2012.00040 22666660 PMC3364462

[6] Dawson MA Gudgin EJ Horton SJ Giotopoulos G Meduri E Robson S. Recurrent mutations, including NPM1c, activate a BRD4-dependent core transcriptional program in acute myeloid leukemia. Leukemia (2014) 28(2):311–20. doi: 10.1038/leu.2013.338 PMC3918873 24220271

[7] El Hajj H Dassouki Z Berthier C Raffoux E Ades L Legrand O. Retinoic acid and arsenic trioxide trigger degradation of mutated NPM1, resulting in apoptosis of AML cells. Blood (2015) 125(22):3447–54. doi: 10.1182/blood-2014-11-612416 25800051

[8] Bienz M Ludwig M Leibundgut EO Mueller BU Ratschiller D Solenthaler M. Risk assessment in patients with acute myeloid leukemia and a normal karyotype. Clin Cancer Res (2005) 11(4):1416–24. doi: 10.1158/1078-0432.CCR-04-1552 15746041

[9] Nitika WJ Hui AM. Role of biomarkers in FLT3 AML. Cancers (Basel) (2022) 14(5):1164. doi: 10.3390/cancers14051164 35267471 PMC8909069

[10] Santos FP Jones D Qiao W Cortes JE Ravandi F Estey EE. Prognostic value of FLT3 mutations among different cytogenetic subgroups in acute myeloid leukemia. Cancer (2011) 117(10):2145–55. doi: 10.1002/cncr.25670 PMC4184429 21523727

[11] Zaidi SZ Owaidah T Al Sharif F Ahmed SY Chaudhri N Aljurf M. The challenge of risk stratification in acute myeloid leukemia with normal karyotype. Hematol Oncol Stem Cell Ther (2008) 1(3):141–58. doi: 10.1016/s1658-3876(08)50023-9 20063545

[12] Ciurea SO Chilkulwar A Saliba RM Chen J Rondon G Patel KP. Prognostic factors influencing survival after allogeneic transplantation for AML/MDS patients with TP53 mutations. Blood (2018) 131(26):2989–92. doi: 10.1182/blood-2018-02-832360 PMC7218750 29769261

[13] Welch JS Petti AA Miller CA Fronick CC O'Laughlin M Fulton RS. TP53 and decitabine in acute myeloid leukemia and myelodysplastic syndromes. N Engl J Med (2016) 375(21):2023–36. doi: 10.1056/NEJMoa1605949 PMC5217532 27959731

[14] Maslah N Salomao N Drevon L Verger E Partouche N Ly P. Synergistic effects of PRIMA-1Met (APR-246) and 5-azacitidine in TP53-mutated myelodysplastic syndromes and acute myeloid leukemia. Haematologica (2020) 105(6):1539–51. doi: 10.3324/haematol.2019.218453 PMC7271596 31488557

[15] Li HY Deng DH Huang Y Ye FH Huang LL Xiao Q. Favorable prognosis of biallelic CEBPA gene mutations in acute myeloid leukemia patients: a meta-analysis. Eur J Haematol (2015) 94(5):439–48. doi: 10.1111/ejh.12450 25227715

[16] Greif PA Dufour A Konstandin NP Ksienzyk B Zellmeier E Tizazu B. GATA2 zinc finger 1 mutations associated with biallelic CEBPA mutations define a unique genetic entity of acute myeloid leukemia. Blood (2012) 120(2):395–403. doi: 10.1182/blood-2012-01-403220 22649106

[17] Hahn CN Chong CE Carmichael CL Wilkins EJ Brautigan PJ Li XC. Heritable GATA2 mutations associated with familial myelodysplastic syndrome and acute myeloid leukemia. Nat Genet (2011) 43(10):1012–7. doi: 10.1038/ng.913 PMC3184204 21892162

[18] De Braekeleer E Douet-Guilbert N De Braekeleer M. RARA fusion genes in acute promyelocytic leukemia: A review. Expert Rev Hematol (2014) 7(3):347–57. doi: 10.1586/17474086.2014.903794 24720386

[19] Noguera NI Catalano G Banella C Divona M Faraoni I Ottone T. Acute promyelocytic leukemia: Update on the mechanisms of leukemogenesis, resistance and on innovative treatment strategies. Cancers (Basel) (2019) 11(10):1591. doi: 10.3390/cancers11101591 31635329 PMC6826966

[20] Nagpal S Friant S Nakshatri H Chambon P. RARs and RXRs: evidence for two autonomous transactivation functions (AF-1 and AF-2) and heterodimerization *in vivo* . EMBO J (1993) 12(6):2349–60. doi: 10.1002/j.1460-2075.1993.tb05889.x PMC413465 8389696

[21] Jimenez JJ Chale RS Abad AC Schally AV. Acute promyelocytic leukemia (APL): a review of the literature. Oncotarget (2020) 11(11):992–1003. doi: 10.18632/oncotarget.27513 32215187 PMC7082115

[22] Ma H Yang J. Insights into the all-trans-Retinoic acid and arsenic trioxide combination treatment for acute promyelocytic leukemia: A meta-analysis. Acta Haematol (2015) 134(2):101–8. doi: 10.1159/000369242 25925330

[23] Xin L Wan-jun S Zeng-jun L Yao-zhong Z Yun-tao L Yan L. A survival study and prognostic factors analysis on acute promyelocytic leukemia at a single center. Leuk Res (2007) 31(6):765–71. doi: 10.1016/j.leukres.2006.07.028 17007927

[24] Creutzig U Kutny MA Barr R Schlenk RF Ribeiro RC. Acute myelogenous leukemia in adolescents and young adults. Pediatr Blood Cancer (2018) 65(9):e27089. doi: 10.1002/pbc.27089 29667722 PMC6105504

[25] Sanz MA Fenaux P Tallman MS Estey EH Löwenberg B Naoe T. Management of acute promyelocytic leukemia: Updated recommendations from an expert panel of the European LeukemiaNet. Blood (2019) 133:1630–43. doi: 10.1182/blood-2019-01-894980 PMC6509567 30803991

[26] Baba SM Pandith AA Shah ZA Baba RA. Pathogenetic implication of fusion genes in acute promyelocytic leukemia and their diagnostic utility. Clin Genet (2019) 95:41–52. doi: 10.1111/cge.13372 29700805

[27] Liquori A Ibañez M Sargas C Sanz MÁ Barragán E Cervera J. Acute promyelocytic leukemia: A constellation of molecular events around a single PML-RARA fusion gene. Cancers (Basel) (2020) 12(3):624. doi: 10.3390/cancers12030624 32182684 PMC7139833

[28] Mosna F Gottardi M. Stem cell modeling of core binding factor acute myeloid leukemia. Stem Cells Int (2016) 2016:7625827. doi: 10.1155/2016/7625827 26880987 PMC4737463

[29] Gilliland DG. Molecular genetics of human leukemias: new insights into therapy. Semin Hematol (2002) 39(4 Suppl 3):6–11. doi: 10.1053/shem.2002.36921 12447846

[30] Rau RE. Beyond KIT in CBF-AML: chromatin and cohesin. Blood (2016) 127(20):2370–1. doi: 10.1182/blood-2016-03-707083 PMC9278272 27207321

[31] Gulley ML Shea TC Fedoriw Y. Genetic tests to evaluate prognosis and predict therapeutic response in acute myeloid leukemia. J Mol Diagn (2010) 12(1):3–16. doi: 10.2353/jmoldx.2010.090054 19959801 PMC2797712

[32] Lagunas-Rangel FA Chávez-Valencia V Gómez-Guijosa MÁ Cortes-Penagos C. Acute myeloid leukemia-genetic alterations and their clinical prognosis. Int J Hematol Oncol Stem Cell Res (2017) 11(4):328–39. PMC5767295 29340131

[33] Seiter K Htun K Baskind P Liu Z. Acute myeloid leukemia in a father and son with a germline mutation of ASXL1. Biomark Res (2018) 6:7. doi: 10.1186/s40364-018-0121-3 29456859 PMC5809979

[34] Ziemin-van der Poel S McCabe NR Gill HJ Espinosa R 3rd Patel Y Harden A. Identification of a gene, MLL, that spans the breakpoint in 11q23 translocations associated with human leukemias. Proc Natl Acad Sci USA (1991) 88(23):10735–9. doi: 10.1073/pnas.88.23.10735 PMC53005 1720549

[35] Armstrong SA Staunton JE Silverman LB Pieters R den Boer ML Minden MD. MLL translocations specify a distinct gene expression profile that distinguishes a unique leukemia. Nat Genet (2002) 30(1):41–7. doi: 10.1038/ng765 11731795

[36] Menghrajani K Gomez-Arteaga A Madero-Marroquin R Zhang MJ Bo-Subait K Sanchez J. Risk classification at diagnosis predicts post-HCT outcomes in intermediate-, adverse-risk, and KMT2A-rearranged AML. Blood Adv (2022) 6(3):828–47. doi: 10.1182/bloodadvances.2021004881 PMC8945306 34551064

[37] Mann G Attarbaschi A Schrappe M De Lorenzo P Peters C Hann I. Interfant-99 study group. improved outcome with hematopoietic stem cell transplantation in a poor prognostic subgroup of infants with mixed-lineage-leukemia (MLL)-rearranged acute lymphoblastic leukemia: Results from the interfant-99 study. Blood (2010) 116(15):2644–50. doi: 10.1182/blood-2010-03-273532 20592248

[38] Schoch C Schnittger S Klaus M Kern W Hiddemann W Haferlach T. AML with 11q23/MLL abnormalities as defined by the WHO classification: incidence, partner chromosomes, FAB subtype, age distribution, and prognostic impact in an unselected series of 1897 cytogenetically analyzed AML cases. Blood (2003) 102(7):2395–402. doi: 10.1182/blood-2003-02-0434 12805060

[39] Pigneux A Labopin M Maertens J Cordonnier C Volin L Socié G. Acute leukemia working party EBMT. outcome of allogeneic hematopoietic stem-cell transplantation for adult patients with AML and 11q23/MLL rearrangement (MLL-r AML). Leukemia (2015) 29(12):2375–81. doi: 10.1038/leu.2015.143 26082270

[40] Shi A Murai MJ He S Lund G Hartley T Purohit T. Structural insights into inhibition of the bivalent menin-MLL interaction by small molecules in leukemia. Blood (2012) 120(23):4461–9. doi: 10.1182/blood-2012-05-429274 PMC3512226 22936661

[41] Figueroa ME Lugthart S Li Y Erpelinck-Verschueren C Deng X Christos PJ. DNA Methylation signatures identify biologically distinct subtypes in acute myeloid leukemia. Cancer Cell (2010) 17(1):13–27. doi: 10.1016/j.ccr.2009.11.020 20060365 PMC3008568

[42] Yang X Wong MPM Ng RK. Aberrant DNA methylation in acute myeloid leukemia and its clinical implications. Int J Mol Sci (2019) 20(18):4576. doi: 10.3390/ijms20184576 31527484 PMC6770227

[43] Majeti R. Clonal evolution of pre-leukemic hematopoietic stem cells precedes human acute myeloid leukemia. Best Pract Res Clin Haematol (2014) 27(3-4):229–34. doi: 10.1016/j.beha.2014.10.003 PMC5419218 25455271

[44] Shlush LI Zandi S Mitchell A Chen WC Brandwein JM Gupta V. Identification of pre-leukaemic haematopoietic stem cells in acute leukaemia. Nature (2014) 506(7488):328–33. doi: 10.1038/nature13038 PMC4991939 24522528

[45] Rampal R Alkalin A Madzo J Vasanthakumar A Pronier E Patel J. DNA Hydroxymethylation profiling reveals that WT1 mutations result in loss of TET2 function in acute myeloid leukemia. Cell Rep (2014) 9(5):1841–55. doi: 10.1016/j.celrep.2014.11.004 PMC4267494 25482556

[46] Paschka P Schlenk RF Gaidzik VI Habdank M Krönke J Bullinger L. IDH1 and IDH2 mutations are frequent genetic alterations in acute myeloid leukemia and confer adverse prognosis in cytogenetically normal acute myeloid leukemia with NPM1 mutation without FLT3 internal tandem duplication. J Clin Oncol (2010) 28(22):3636–43. doi: 10.1200/JCO.2010.28.3762 20567020

[47] Marcucci G Maharry K Wu YZ Radmacher MD Mrózek K Margeson D. IDH1 and IDH2 gene mutations identify novel molecular subsets within *de novo* cytogenetically normal acute myeloid leukemia: A cancer and leukemia group b study. J Clin Oncol (2010) 28(14):2348–55. doi: 10.1200/JCO.2009.27.3730 PMC2881719 20368543

[48] Patel JP Gönen M Figueroa ME Fernandez H Sun Z Racevskis J. Prognostic relevance of integrated genetic profiling in acute myeloid leukemia. N Engl J Med (2012) 366(12):1079–89. doi: 10.1056/NEJMoa1112304 PMC3545649 22417203

[49] Debarri H Lebon D Roumier C Cheok M Marceau-Renaut A Nibourel O. IDH1/2 but not DNMT3A mutations are suitable targets for minimal residual disease monitoring in acute myeloid leukemia patients: a study by the acute leukemia French association. Oncotarget (2015) 6(39):42345–53. doi: 10.18632/oncotarget.5645 PMC4747230 26486081

[50] Prakash C Fan B Altaf S Agresta S Liu H Yang H. Pharmacokinetics, absorption, metabolism, and excretion of [14C]ivosidenib (AG-120) in healthy male subjects. Cancer Chemother Pharmacol (2019) 83(5):837–48. doi: 10.1007/s00280-019-03793-7 30758648

[51] Dutta R Zhang TY Köhnke T Thomas D Linde M Gars E. Enasidenib drives human erythroid differentiation independently of isocitrate dehydrogenase 2. J Clin Invest (2020) 130(4):1843–9. doi: 10.1172/JCI133344 PMC7108889 31895700

[52] Ma QL Wang JH Yang M Wang HP Jin J. MiR-362-5p as a novel prognostic predictor of cytogenetically normal acute myeloid leukemia. J Transl Med (2018) 16(1):68. doi: 10.1186/s12967-018-1445-3 29540187 PMC5853092

[53] Zhang TJ Wu DH Zhou JD Li XX Zhang W Guo H. Overexpression of miR-216b: Prognostic and predictive value in acute myeloid leukemia. J Cell Physiol (2018) 233(4):3274–81. doi: 10.1002/jcp.26171 28884855

[54] Stark MS Tyagi S Nancarrow DJ Boyle GM Cook AL Whiteman DC. Characterization of the melanoma miRNAome by deep sequencing. PloS One (2010) 5(3):e9685. doi: 10.1371/journal.pone.0009685 20300190 PMC2837346

[55] Eisfeld AK Marcucci G Maharry K Schwind S Radmacher MD Nicolet D. miR-3151 interplays with its host gene BAALC and independently affects outcome of patients with cytogenetically normal acute myeloid leukemia. Blood (2012) 120(2):249–58. doi: 10.1182/blood-2012-02-408492 PMC3398762 22529287

[56] Díaz-Beyá M Brunet S Nomdedéu J Cordeiro A Tormo M Escoda L. The expression level of BAALC-associated microRNA miR-3151 is an independent prognostic factor in younger patients with cytogenetic intermediate-risk acute myeloid leukemia. Blood Cancer J (2015) 5(10):e352. doi: 10.1038/bcj.2015.76 26430723 PMC4635188

[57] Díaz-Beyá M Brunet S Nomdedéu J Tejero R Díaz T Pratcorona M. MicroRNA expression at diagnosis adds relevant prognostic information to molecular categorization in patients with intermediate-risk cytogenetic acute myeloid leukemia. Leukemia (2014) 28(4):804–12. doi: 10.1038/leu.2013.281 24072101

[58] Lin X Wang Z Zhang R Feng W. High serum microRNA-335 level predicts aggressive tumor progression and unfavorable prognosis in pediatric acute myeloid leukemia. Clin Transl Oncol (2015) 17(5):358–64. doi: 10.1007/s12094-014-1237-z 25301405

[59] Zhao J Lu Q Zhu J Fu J Chen YX. Prognostic value of miR-96 in patients with acute myeloid leukemia. Diagn Pathol (2014) 9:76. doi: 10.1186/1746-1596-9-76 24678958 PMC3975266

[60] Butrym A Rybka J Baczyńska D Tukiendorf A Kuliczkowski K Mazur G. Low expression of microRNA-204 (miR-204) is associated with poor clinical outcome of acute myeloid leukemia (AML) patients. J Exp Clin Cancer Res (2015) 34(1):68. doi: 10.1186/s13046-015-0184-z 26126974 PMC4508825

[61] Abdelhafiz AS Elsayed GM Saber MM Gameel A Hamdy N. Low expression of miR-204 is associated with expression of CD34 and poor performance status in denovo AML. Int J Lab Hematol (2020) 42(3):263–9. doi: 10.1111/ijlh.13161 32048789

[62] Hu N Cheng Z Pang Y Zhao H Chen L Wang C. High expression of MiR-98 is a good prognostic factor in acute myeloid leukemia patients treated with chemotherapy alone. J Cancer (2019) 10(1):178–85. doi: 10.7150/jca.26391 PMC6329859 30662538

